# A new comprehensive trait database of European and Maghreb butterflies, Papilionoidea

**DOI:** 10.1038/s41597-020-00697-7

**Published:** 2020-10-15

**Authors:** Joseph Middleton-Welling, Leonardo Dapporto, Enrique García-Barros, Martin Wiemers, Piotr Nowicki, Elisa Plazio, Simona Bonelli, Michele Zaccagno, Martina Šašić, Jana Liparova, Oliver Schweiger, Alexander Harpke, Martin Musche, Josef Settele, Reto Schmucki, Tim Shreeve

**Affiliations:** 1 Centre for Ecology, Environment and Conservation, Faculty of Health and Life Sciences, Oxford Brookes University, Oxford, OX3 0BP UK; 2 ZEN lab., Dipartimento di Biologia dell’Università di Firenze, Via Madonna del Piano 6, 50019 Comune di Sesto Fiorentino, Firenze, Italy; 3 Department of Biology, Universidad Autónoma de Madrid, Campus Cantoblanco, 28049 Madrid, Spain; 4 Senckenberg Deutsches Entomologisches Institut, Eberswalder Str. 90, 15374 Müncheberg, Germany; 5 Department of Community Ecology, Helmholtz Centre for Environmental Research - UFZ, Theodor-Lieser-Strasse 4, 06120 Halle, Germany; 6 Institute of Environmental Sciences, Jagiellonian University, Gronostajowa 7, 30-387 Kraków, Poland; 7 Department of Life Sciences and Systems Biology, Università degli Studi di Torino, Via Accademia Albertina 13, 10123 Torino, Italy; 8 Croatian Natural History Museum, Demetrova 1, 10 000 Zagreb, Croatia; 9 Institute of Entomology, Biology Centre CAS, Branisovska 31, Ceske Budejovice, Czech Republic; 10 iDiv, German Centre for Integrative Biodiversity Research, Halle-Jena-Leipzig, Deutscher Platz 5e, 04103 Leipzig, Germany; 11 UK Centre for Ecology and Hydrology, Maclean Building, Benson Lane, Crowmarsh Gifford, Wallingford, OX10 8BB UK

**Keywords:** Conservation biology, Entomology, Biodiversity

## Abstract

Trait-based analyses explaining the different responses of species and communities to environmental changes are increasing in frequency. European butterflies are an indicator group that responds rapidly to environmental changes with extensive citizen science contributions to documenting changes of abundance and distribution. Species traits have been used to explain long- and short-term responses to climate, land-use and vegetation changes. Studies are often characterised by limited trait sets being used, with risks that the relative roles of different traits are not fully explored. Butterfly trait information is dispersed amongst various sources and descriptions sometimes differ between sources. We have therefore drawn together multiple information sets to provide a comprehensive trait database covering 542 taxa and 25 traits described by 217 variables and sub-states of the butterflies of Europe and Maghreb (northwest Africa) which should serve for improved trait-based ecological, conservation-related, phylogeographic and evolutionary studies of this group of insects. We provide this data in two forms; the basic data and as processed continuous and multinomial data, to enhance its potential usage.

## Abstract (summary)

**Table:** 

Measurement(s)	resources • Egg Laying • larval environment • pupal environment • geographic location • behavior • size • voltinism • phenology • host plant
Technology Type(s)	digital curation
Factor Type(s)	species
Sample Characteristic - Organism	Papilionoidea
Sample Characteristic - Location	Europe • Northwest Africa

Machine-accessible metadata file describing the reported data: 10.6084/m9.figshare.12998828

## Background & Summary

The taxonomy, distribution, and biology of European butterflies has been studied since the 18^th^ century. Due to the precise knowledge of changes in distribution and abundance, driven by extensive citizen science contributions, and their trophic specialisation and immediate responses to environmental changes^1^ they are frequently used as indicators of environmental change^2^. Recently, a series of comprehensive resources have been published for European butterflies comprising a detailed taxonomic list^3^, a dataset for 15,609 sequences for the COI mitochondrial markers for all Western-Central European species^4^, a dated phylogenetic tree for all European species^5^, atlases describing their detailed distributions^6^, and climatic risk assessments^7^. In turn, species traits are fundamental descriptors of feeding ecology, life-history, morphology, resource use, behaviour and physiological constraints^8^. It has long been recognised^9^ that the availability of such data is largely limited and incomplete. Only recently has a geographically extensive series of traits describing climatic preferences based on temperature and precipitation been produced^10^ along with a series of six traits describing some features of feeding ecology, morphology, and life histories of butterflies from Western and Central Europe^4^. Species traits have been used to explain extinction risk and conservation status^11–13^, colonisation and distribution changes^14,15^, phenology and potential for range shifts in relation to climate change^16–18^ and phylogeographic patterns^4^ over large spatial scales. More detailed trait information at smaller spatial scales has been used to identify ecological groupings of species, for both butterflies in the British Isles^19^ and macromoths of Central Europe^20^. However, most studies which have used trait information to identify ecological relationships, current extinction risks and distributions have tended to use either limited sources of information and/or limited numbers of butterfly species or have been concentrated at the regional scale^21,22^.

Because single sources of trait information may be limited (e.g. in geographical scope) or conflict with each other^23^, we present a new comprehensive open-access trait database^24^^,^ with a maintained version (https://butterflytraits.github.io/European-Butterfly-Traits/index.html) of the European and Maghreb butterflies. We have aimed to pull together all the existing trait information available for each species. This has been done by synthesising the existing information from field guides, ecological atlases, reliable on-line sources, expert opinion and journal articles. Our database provides trait information for 542 taxa and covers 25 main traits (some subdivided - giving 217 trait states in total), including life history, resource use by all life-cycle stages, and behavioural information. Where specific traits are variable within species we also give data on this variability. We also process this data to provide multinomial and continuous variables and measures of their variability, resulting in a matrix of 542 species by 31 variables. We also list our information sources for the traits. Although some previous trait-based analyses have included vegetation associations, our trait database does not include these for two reasons. First, the habitat a species occurs in is determined by the occurrence and spatial distributions of species-specific resources^25,26^. Second, resource, life-history and behavioural traits can be used to predict the vegetation structures in which species occur^19^.

Our database provides an outstanding resource for improving our understanding of fundamental mechanisms and processes such as how traits define species occurrence and co-occurrences, their responses to environmental change, their spatial dynamics, and their associations with vegetation structures. Since traits vary within different taxonomic groups, understanding their evolution and variability within different branches of the tree of life can also provide insights into phylogenetic constraints on species resource requirements and ultimately on their local abundance and large-scale occurrence and vulnerability to environmental change.

## Methods

### Taxon and geographic coverage

**Fig. 1:**
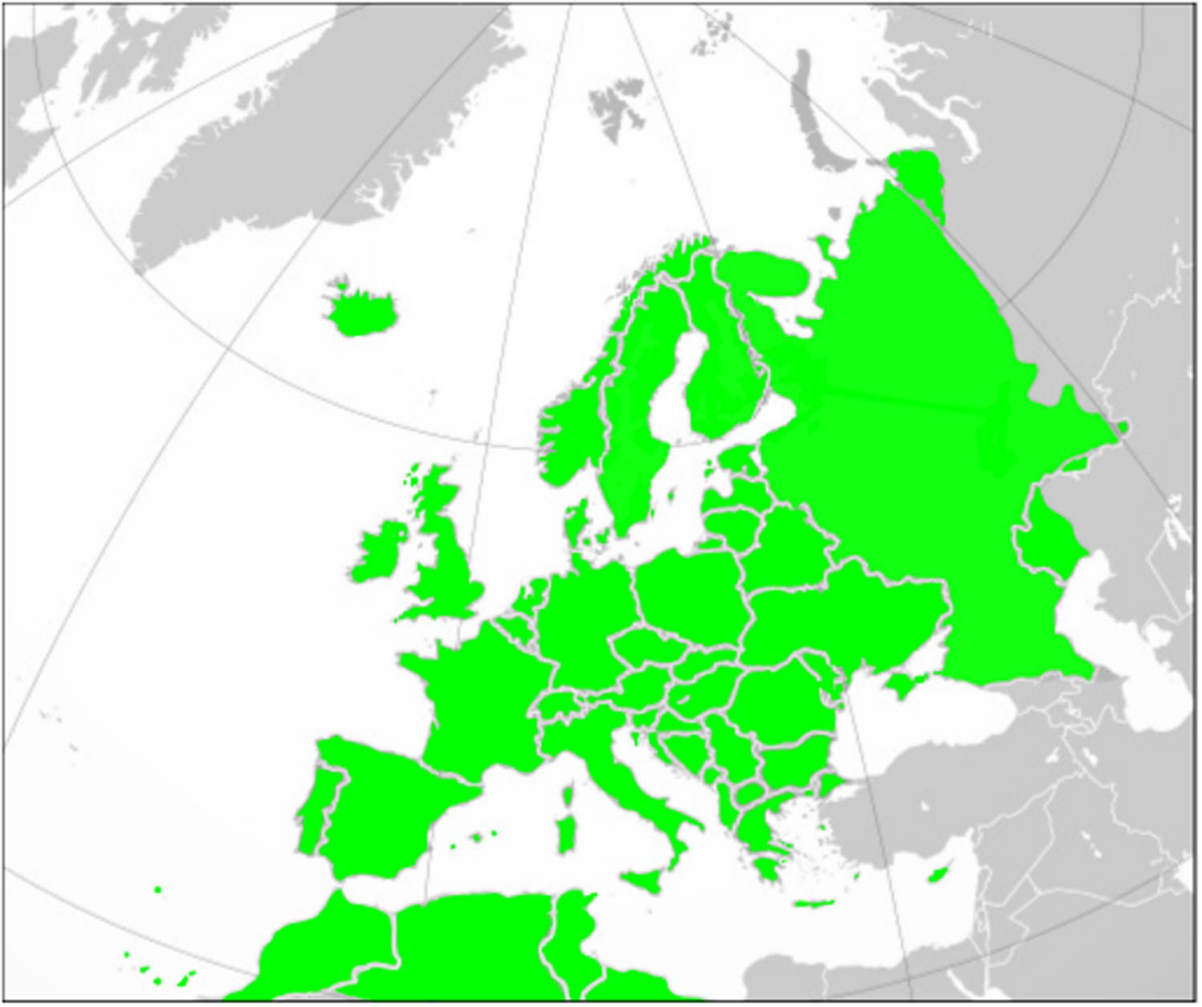
The geographic area covered by the European and Maghreb butterfly database.

Our dataset (542 taxa) represents the complete butterfly fauna of mainland Europe including the western parts of Russia, the European islands, Macaronesia and the Maghreb (North Africa). This includes all of the 496 species occurring in Europe according to the latest checklist of European butterflies^3^, but we have also included taxa (Table 1) that are confined the Maghreb or have very divergent traits from the nominate species according to some sources within our study area (Fig. 1). The nomenclature is consistent with that used in the checklist^3^. Trait information is recorded for all the families of butterflies included in the geographic area (Papilionidae, Hesperiidae, Pieridae, Riodinidae, Lycaenidae and Nymphalidae). For species that also occur beyond the study area, trait information was taken from the main study area, if possible. For example, for those species that have a pan-Palearctic distribution only information from the European range was included in our dataset.

**Table 1:** Species not in the European checklist^3^ but included in the trait database of European and Maghreb butterflies.

Species	Reason
*Anthocharis belia*	Non-European (Maghreb Endemic)
*Argynnis auresiana*	Non-European (Maghreb Endemic)
*Berberia Abdelkader*	Non-European (Maghreb Endemic)
*Berberia lambessanus*	Non-European (Maghreb Endemic)
*Cigaritis allardi*	Non-European (Maghreb Endemic)
*Cigaritis siphax*	Non-European (Maghreb Endemic)
*Cigaritis zohra*	Non-European (Maghreb Endemic)
*Coenonympha arcanioides*	Non-European (Maghreb Endemic)
*Coenonympha corinna elbana*	Sub-species
*Coenonympha fettigii*	Non-European (Maghreb Endemic)
*Coenonympha gardetta darwiniana*	Sub-species
*Coenonympha vaucheri*	Non-European (Maghreb Endemic)
*Cupido minimus carswelli*	Sub-species
*Deudorix livia*	Very small (possibly doubtful) European range
*Euchloe falloui*	Non-European (N Africa & SW Asia)
*Hipparchia algirica*	Non-European (Maghreb Endemic)
*Hipparchia azorina occidentalis*	Sub-species
*Hipparchia caroli*	Non-European (Maghreb Endemic)
*Hipparchia ellena*	Non-European (Maghreb Endemic)
*Hipparchia genava*	Disputed species
*Hipparchia hansii*	Non-European (Maghreb Endemic)
*Hipparchia powelli*	Non-European (Maghreb Endemic)
*Hyponephele maroccana*	Non-European (Maghreb Endemic)
*Lasiommata meadewaldoi*	Non-European (Maghreb Endemic)
*Lycaena phoebus*	Non-European (Maghreb Endemic)
*Lysandra punctifera*	Non-European (Maghreb Endemic)
*Melanargia lucasi*	Non-European (Maghreb Endemic)
*Melitaea deserticola*	Non-European (Maghreb Endemic)
*Muschampia mohammed*	Non-European (Maghreb Endemic)
*Papilio saharae*	Non-European (N Africa & SW Asia)
*Pieris segonzaci*	Non-European (Maghreb Endemic)
*Plebejus allardii*	Non-European (Maghreb Endemic)
*Plebejus martini*	Non-European (Maghreb Endemic)
*Plebejus vogelii*	Non-European (Maghreb Endemic)
*Polyommatus atlanticus*	Non-European (Maghreb Endemic)
*Polyommatus dolus virgilius*	Sub-species
*Polyommatus icarus andronicus*	Sub-species
*Pontia glauconome*	Non-European (N Africa & SW Asia)
*Pseudochazara atlantis*	Non-European (Maghreb Endemic)
*Pseudochazara mamurra*	Non-European in southern Balkans
*Pseudochazara mniszechii*	Non-European in southern Balkans
*Pseudophilotes fatma*	Non-European (Maghreb Endemic)
*Pyronia janiroides*	Non-European (Maghreb Endemic)
*Tarucus rosaceus*	Non-European (Maghreb Endemic)
*Thymelicus hamza*	Non-European (Maghreb Endemic)
*Tomares mauretanicus*	Non-European (Maghreb Endemic)

Trait information was gathered from sources including field guides, books and atlases^27–62^, scientific papers^63–166^, and some selected online resources (https://butterfliesoffrance.com, https://iucnredlist.org, https://lepidoptera.sk, http://luontoportti.com/suomi/enhttp://leps.it, http://eurobutterflies.comhttp://www.lepiforum.de, https://minambiente.it/home_naturahttps://micheltarrier.com/micheltarrier-com/rhopalocera, http://babochki-kavkaza.ru, http://pyrgus.de, http://butterflyeurope.co.uk, http://lepiforum.de) and direct observation in the field. Species-specific information sources are given in the database^24^ and website (https://butterflytraits.github.io/European-Butterfly-Traits/index.html). In cases with multiple sources of trait information, data from peer reviewed papers were preferentially used; in practice this made up a small proportion of the total trait data. In cases where differences were identified in trait information between different sources, and could be identified as representing trait diversity, all sets of information were included in the trait database. Where sources clearly conflicted, we used the information that we deemed the most reliable. When published information was lacking, we inferred traits using photographs from two reliable sources (https://www.leps.it & http://pyrgus.de) if the traits could be unequivocally determined. This included hostplants and hostplant types, egg-laying location, larval location, adult feeding, adult basking type and basking sites. Trait information based on photographs was independently assessed by the authors in order to check the validity of the inferences. For some taxa certain trait information was not available in any source, and thus it is missing in the database.

The first version of the trait database^24^ was finalised after three steps had been completed for all taxa. These were 1) mining all the standard references (e.g guides and atlases) for trait information, 2) filling gaps in trait information through a thorough literature search via Google Scholar and PubMed, and 3) emailing and asking experts on particular taxa for additional trait information.

### Trait types

Our database covers the traits of all stages of the butterfly life cycle. Many trait types included in the database along with their subdivisions into individual states were derived from an earlier treatment of the butterflies of the British Isles^19^ but the trait types have been extended for this database. Individual traits were defined prior to the beginning of data collation to allow for unambiguous coding. Comprehensive trait definitions are in the file traitdefinitions.pdf on the Dryad repository^24^ and curated on-line version (https://butterflytraits.github.io/European-Butterfly-Traits/index.html). Most of the traits types in our raw data trait database (state table) are coded as binary, variables, but a minority are continuous (Online-only Table 1). Most traits included in this dataset are divided into multiple sub-traits. For example, the trait ‘overwintering stage’ comprises four binary sub-traits each of which indicates one stage of a species’ life cycle: egg, larva, pupa or adult. A species can have any combination of 0 and 1 for each of these sub-traits. This allows for the coding of trait plasticity across a species’ range. Likewise, voltinism is coded as binary values for different states. This coding of the basic data (state table) has been transformed (traits table) into a series of multinomial traits, derived from binary states in the state table, and as continuous data, where the data in the state table is also continuous (Online-only Table 2). Additional variables are added in the traits table to describe variability within traits, resulting in a matrix of 542 species x 31 trait variables. Presenting the basic data within the database (state table) facilitates adding data in the future, which could include novel combinations of sub-trait states, whilst providing the processed data (traits table) and original raw data (state table) aids different analytical procedures.

Traits are divided into four main types: ‘life history’, ‘morphological’,‘resource-based’, and ‘behavioural’. Life history traits describe a species’ life cycle related to reproduction and also to growth and survival, including the number of generations per year (voltinism), egg laying strategy (egg laying type) and overwintering stage. We use wing size (both forewing length and precisely defined wingspan) as key morphological traits because they have been used in previous trait-based analyses, being correlated with mobility^167–169^, development time^170^, and reproductive output^171^, as size correlates with many aspects of life history^172,173^. Wingspan is included as it also includes an approximate measure of thoracic size, and thus flight muscle mass which may influence flight capacity and dynamics. ‘Resource-based traits’ describe species’ relationships with environmental resources. Resources include consumables that can be depleted over time when used or utilities that are not depleted. For example, ‘adult feeding’ describes the range of resources consumed by adults which may be temporarily or permanently depleted. Likewise, ‘adult roosting’ describes structures (utilities) used for roosting behaviour; these structures are resources, and although not directly consumed there may be a finite number of suitable features of this type within a location which may be limiting factors for local populations and may become the subject of both interspecific and intraspecific competition^25,26^.

Some traits in the database are primarily behavioural such as ‘mate locating type’, but these traits are also closely linked with traits that relate more directly to resource-usage (in this case with ‘mate locating location’); thus, behavioural traits can also be linked to resources. Larval hostplants are examined in detail in several traits because of their importance for the life cycle and population structure of butterflies. Some authors of previous work using butterfly traits have included ‘habitat breadth’ as a trait^14,174^, although the physical structures/vegetation types occupied by species are not traits themselves, but the result of species occurring in those locations where their essential resources co-occur in spatial patterns and densities that they can use and these can change substantially across the geographic area our database covers. Essentially, species habitats are defined by their resources^25,26^ and the resource requirements that species have are fundamental traits. Biotope or habitat associations are therefore not included in this dataset as they can be derived from the traits described in our database^24^. Additionally, biotope traits have been shown to have poor reproducibility among different trait sources^23^ and have been found to be less useful than other types of traits for understanding the responses of butterflies to environmental change over time at a large scale^19,23^. At a smaller scale, biotope associations may be useful characteristics for aiding in butterfly conservation and habitat classification, but any attempt to synthesise information at a large geographic scale describing habitat preferences from multiple sources would likely be both error-prone and probably too coarse for most analyses. We also did not include measures of climatic requirements and geographic ranges in our dataset since they are already publicly available in the CLIMBER dataset^10^.

## Data Records

The database^24^ deposited on the Dryad Digital Repository and the live version (https://butterflytraits.github.io/European-Butterfly-Traits/index.html) including species specific information sources and a PDF-file describing each of the variables in the raw state table and traits table (ButterflyTraitDefinitions.pdf). The live version includes a mechanism for feedback and adding new information. For some taxa there are missing data and some traits currently have more missing values than others. Life history and hostplant related traits are extensively covered with few missing values, but behavioural traits have the most missing values as they usually require direct observation in the field, thus the disparity. However, the types of traits with missing data (Table 2) indicate where targeted fieldwork is required. Likewise, species with poor overall data also warrant targeted future effort.

**Table 2:** The percentage of each family within the European and Maghreb butterflies trait database with incomplete trait data, described by 31 multinomial and continuous variables in the traits table.

Trait	Percentage of species within families with missing trait data						All (n = 542)
	Papiionidae (n = 16)	Hesperiidae (n = 49)	Pieridae (n = 61)	Riodinidae (n = 1)	Lycaenidae (n = 146)	Nymphalidae (n = 269)	
Overwintering stage	0	16.3	4.9	0	17.8	15.6	14.6
Overwintering location	12.5	40.8	24.6	0	37.0	54.3	43.7
Pupal location	18.8	36.7	19.7	0	38.4	40.1	36.3
Voltinism minimum	0	0	0	0	0	0.4	0.2
Voltinism maximum	0	0	0	0	0	0.4	0.2
Ant association	0	0	0	0	39.4	1.5	11.3
Wing index	0	0	0	0	0	0	0
Wing index variation	0	0	0	0	0	0	0
Hostplant specificity	0	4.1	1.7	0	4.8	15.9	9.8
Hostplant trophic category	0	4.1	0	0	3.4	15.9	9.2
Hostplant specificity index	6.3	8.2	6.6	0	9.6	11.9	10.1
Hostplant type	6.3	10.2	9.8	0	17.1	38.3	25.8
Hostplant growth form	6.3	10.2	9.8	0	17.8	36.1	24.9
Hostplant part	25.0	36.7	21.3	0	32.9	52.0	41.1
Hostplant age	43.8	67.3	41.0	0	74.6	81.0	72.3
Larval environment	0	36.7	21.3	0	28.1	40.9	33.6
Hostplant patch size	93.8	79.6	91.8	0	85.6	86.6	86.3
Egg laying type	6.25	36.7	14.8	0	37.7	43.9	37.1
Egg laying location	12.5	34.7	14.8	0	22.6	37.2	29.7
Egg laying light environment	75	75.5	82	0	77.4	82.2	79.9
Flight months min., max., av.	0	0	0	0	0	0	0
First and last flight months	0	0	0	0	0	0	0
Adult feeding	0	16,3	14.8	0	21.2	25.7	21.6
Adult roosting	37.5	81.6	44.3	0	75.3	68.0	67.5
Mate locating type	18.8	63.3	27.9	0	65.1	66.1	59.8
Mate locating location	18.8	67.3	32.8	0	76.0	73.6	67.3
Basking type	0	44.9	27.8	0	53.4	42.4	42.6
Basking location	0	44.9	32.8	0	51.4	43.1	43.0

## Technical Validation

The records included in the database are based on previously published information from field guides, ecological atlases and peer reviewed journal articles, supplemented with the authors’ personal observations. We are therefore confident as to their accuracy. When sources highlighted that records for a particular trait were doubtful, this information was not included in the dataset. The author team comprises experts on butterfly ecology coming from seven countries across Europe thus ensuring the highest level of repeated quality control while providing best knowledge across the biomes in Europe. The authors have examined the dataset to check for errors and to assess the accuracy of the trait information included. All data included in the dataset is fully referenced which allows anyone to go back to the original records for any piece of trait information. The dataset currently contains some missing values, especially for highly localised species and we intend to keep the database ‘live’ and to manage updates with new information. Certain traits such as voltinism and phenology (flight months) are known to vary across the latitudinal gradient as these traits may in part be responses to accumulated growing degree days^175^. We are confident that we have captured variability of these traits for the majority of species by consulting trait sources that encompass both the full European range as well as smaller areas. We will accept data into our live version of the database from existing resources, unpublished information and new published information. Each species has its own reference list so existing data can be checked and new information correctly integrated into the database. Data submission methods are described in the live database.

## Usage Notes

We have provided the first extensive database of butterfly traits in Europe and North Africa. Of particular value is the species and geographic coverage and the extensive sets of traits that we have included. This provides an outstanding resource for improving our understanding of fundamental processes such as how traits define species co-occurrences and their responses to environmental change, their spatial dynamics, and their associations with vegetation structures. Since traits vary within different taxonomic groups, understanding their evolution and variability among different branches of the tree of life can also provide insights into phylogenetic constraints on species resource requirements and ultimately on their local abundance and large-scale occurrence and vulnerability to environmental change. As our trait database includes a large component of resource requirements for all life-history stages it can also be used to aid conservation efforts by focusing on resources that may be limited for vulnerable species at small to large spatial scales. Additionally, the inclusion of behavioural traits within the database can contribute to increasing our understanding of the roles of behavioural characteristics in determining species occurrences and resource use.

We have minimised processing of the data within the state table of the database. Individual variables in this state table may have poor linear relationships or spurious negative correlations due to their statistical distributions and outlier effects which can constrain both phylogenetic and ecological analyses. Although fuzzy methods of multivariate analyses may accommodate these issues^176^ the processed multinomial and continuous variables with measures of variability provided in the traits table facilitates more conventional approaches to multivariate analyses. For some species there is missing data and some traits currently have more missing values than others. Whilst updating the database will supply some missing data there are imputation methods^177,178^ that can be used to predict these values and we are confident that in the absence of verified data, imputed data can be used to retain both species and traits with missing values within analyses.

## Online-only Tables

**Online-only Table 1:** Traits included in the raw ‘state table’ of the database for the European and Maghreb butterflies.

Trait	Coding	Trait type	Sub-trait categories
Overwintering stage (OvS)	Binary	Life history	Egg (E), larva (L), pupa (P), adult (A)
Overwintering location (OvL)	Binary	Resource	Buried (Bu), surface (Su), short sward (Ss), tall sward (Ts), shrub (Sb), tree (Tr), liana (Li)
Voltinism (Vol)	Binary	Life history	Biennial (0.5), univoltine (1), univoltine + partial generation (1.5), bivoltine (2), multivoltine (3)
Pupal location (PuL)	Binary	Resource	Buried (Bu), ground layer (Gl), field layer (Fl), shrub layer (Sl), canopy (Cl), attended by ants (At)
Ant association (AnA)	Binary	Resource	Monospecific (one species) (M), oligospecific (within one genus) (O), polyspecific (multiple genera) (P)
Forewing length (FoL)	Continuous	Morphological	Male average, male minimum, male maximum, male variance. Female average, female minimum, female maximum, female variance. Data obtained from various sources (var), from Higgins and Riley (1970) (HR) and from Tennents (1996) (Ten).
Wingspan (WSp)	Continuous	Morphological	Male average, male minimum, male maximum, male variance. Female average, female minimum, female maximum, female variance
Hostplant family (HPF)	Binary	Resource	70 hostplant families
Hostplant specificity(HPS)	Binary	Resource	Monophagous, oligophagous, polyphagous.
Hostplant type (HPT)	Binary	Resource	Biennial (Bi), annual (An), herbaceous perennial (Hp), woody perennial (Wp), combined perennial (Pe)
Hostplant growth form (HPG)	Binary	Resource	Short herb/grass (<1 m) (Sh), tall herb/grass (>1 m) (Th), shrub (Sb), tree (Tr), liana (Li)
Hostplant part (HPP)	Binary	Resource	Flowers/pod (Fl), leaf (Le), bud (Bu), stem (St)
Hostplant age (HPA)	Binary	Resource	Small/immature (Si), old (Ol)
Larval environment (LEv)	Binary	Resource	Buried (Bu), ground layer (Gl), field layer (Fl), shrub layer (Sl), canopy (Cl), attended by ants (At)
Hostplant patch size (HPSi)	Binary	Resource	Large patch (Lp), medium patch (Mp), small patch/single plant (Ss)
Egg laying type (ELT)	Binary	Life history	Single (Se), small batch ( ≤ 8) (Sb), large batch (>8) (Lb)
Egg laying location (ELL)	Binary	Resource	Bare ground (Bg), short turf/herbs/grass (<1 m) (Sh), tall herbs/grass (>1 m) (Th), shrub (Sb), tree trunk (Tt), canopy Ca), liana (Li)
Egg laying aspect (ELA)	Binary	Resource	Ligh (Li), partial shade (Ps), shade (Sh)
Flight months (FMo)	Binary and Continuous	Life history	Binary: January to December
Adult feeding (AdF)	Binary	Resource	Herb flower (Hf), ergot (Er), shrub/tree flower (Sf), honeydew (Hd), sap (Sa), decaying plant (Dp), animal (An), mineral (Mi)
Adult roosting (ArR)	Binary	Behaviour/Resource	Bare ground (Bg), tree trunk (Tt), short turf (St), grasses (Gr), short herb (<1 m) (Sh), tall herb (>1 m) (Th), shrub (Sb), tree canopy (Tc), liana (Li), exposed (Ex), communal (Co), off-hostplant (Hf), on-hostplant (Hn), man-made structure (Ms)
Mate locating type (MLT)	Binary	Behaviour	Patrolling (Pa), perching (non-territorial) (Pn), perching (territorial) (Pt), lekking (Le)
Mate locating location (MLL)	Binary	Resource/Behaviour	Rock/cliff (Rc), bare earth (Be), short herbs (<1 m) (Sh), grass (Gr), tall herbs (>1 m) (Th), shrubs (Sb), tree canopy (Tc), nectar site (Ns), hostplant site (Hs), physical edge site (Ps), light edge site (Ls), hilltop (Ht)
Basking type (BaT)	Binary	Resource/Behaviour	Dorsal absorption (Da), dorsal reflectance (Dr), lateral (La)
Basking site (BaS)	Binary	Resource	Bare ground (Bg), grass (Gr), short herb (>1 m) (Sh), tall herb (<1 m) (Th), shrub (Sb), tree (Tr), man-made structure (Ms)

Trait coding shows whether each trait is represented as a series of binary sub-traits, or as continuous values. Most traits are made up of a number of smaller sub-traits.

**Online-only Table 2:** The 31 trait states included in the coded trait database.(traits table).

Trait	Coding	Trait type	Sub-trait and categories
Overwintering stage (OvS)	Multinomial	Life history	Egg (E), larva (L), pupa (P), adult (A)
Overwintering location (OvL)	Multinomial	Resource	Buried (Bu), surface (Su), short sward (Ss), tall sward (Ts), shrub (Sb), tree (Tr), liana (Li)
Pupal location (PuL)	Multinomial	Resource	Buried (Bu), ground layer (Gl), field layer (Fl), shrub layer (Sl), canopy (Cl), attended by ants (At)
Voltinism (Vol_min, Vol_max)	Continuous	Life history	Minimum (Vol_min) and maximum (Vol_max) number of generations per year. Biennial (0.5), univoltine (1), univoltine + partial generation (1.5), bivoltine (2), multivoltine (3)
Ant association (AnA)	Multinomial	Resource	Monospecific (one species) (M), oligospecific (within one genus) (O), polyspecific (multiple genera) (P)
Wing index (WIn)	Continuous	Morphological	Calculated index of wingspan and forewing length
Wing index variation (WIV)	Continuous	Morphological	Calculated index of variation in wingspan and forewing length
Hostplant specificity (HPS, TrC, HSI)	Continuous/multinomial	Resource	Total genera of hostplants (HPS), Trophic_category (TrC), monophagous (M), oligophagous (O), polyphagous (P); Hostplant specificity index (HSI)
Hostplant type (HPT)	Multinomial	Resource	Biennial (Bi), annual (An), herbaceous perennial (Hp), woody perennial (Wp)
Hostplant growth form (HPG)	Multinomial	Resource	Short herb/grass (<1 m) (Sh), tall herb/grass (>1 m) (Th), shrub (Sb), tree (Tr), liana (Li)
Hostplant part (HPP)	Multinomial	Resource	Flowers/pod (Fl), leaf (Le), bud (Bu), stem (St), non-plant (Np)
Hostplant age (HPA)	Binomial	Resource	Small/immature (Si), old (Ol)
Larval environment (LEv)	Multinomial	Resource	Buried (Bu), ground layer (Gl), field layer (Fl), shrub layer (Sl), canopy (Cl), attended by ants (At)
Hostplant patch size (HPSi)	Multinomial	Resource	Large patch (Lp), medium patch (Mp), small patch/single plant (Ss)
Egg laying type (ELT)	Multinomial	Life history	Single (Se), small batch (≤8) (Sb), large batch (>8) (Lb)
Egg laying location (ELL)	Multinomial	Resource	Bare ground (Bg), short turf/herbs/grass (<1 m) (Sh), tall herbs/grass (>1 m) (Th), shrub (Sb), tree trunk (Tt), canopy Ca), liana (Li)
Egg laying aspect (ELA)	Multinomial	Resource	Light (Li), partial shade (Ps), shade (Sh)
Flight months (FMo_min, FMo_max, FMo_average, FFM, LFM)	Continuous	Life history	Minimum (FMo_min) and maximum (FMo_max) duration of yearly flight period; average between maximum and minimum duration (FMo_average); first flight month (FFM); last flight month (LFM)
Adult feeding (AdF)	Multinomial	Resource	Herb flower (Hf), ergot (Er), shrub/tree flower (Sf), honeydew (Hd), sap (Sa), decaying plant (Dp), animal (An), mineral (Mi)
Adult roosting (AdR)	Multinomial	Behaviour/Resource	Bare ground (Bg), tree trunk (Tt), short turf (St), grasses (Gr), short herb (<1 m) (Sh), tall herb (>1 m) (Th), shrub (Sb), tree canopy (Tc), liana (Li), exposed (Ex), communal (Co), off-hostplant (Hf), on-hostplant (Hn), man-made structure (Ms)
Mate locating type (MLT)	Multinomial	Behaviour	Patrolling (Pa), perching (non-territorial) (Pn), perching (territorial) (Pt), lekking (Le)
Mate locating location (MLL)	Multinomial	Resource/Behaviour	Rock/cliff (Rc), bare earth (Be), short herbs (<1 m) (Sh), grass (Gr), tall herbs (>1 m) (Th), shrubs (Sb), tree canopy (Tc), nectar site (Ns), hostplant site (Hs), physical edge site (Ps), light edge site (Ls), hilltop (Ht)
Basking type (BaT)	Multinomial	Resource/Behaviour	Dorsal absorption (Da), dorsal reflectance (Dr), lateral (La)
Basking site (BaS)	Multinomial	Resource	Bare ground (Bg), grass (Gr), short herb (>1 m) (Sh), tall herb (<1 m) (Th), shrub (Sb), tree (Tr), man-made structure (Ms)

Traits coded with a series of binary variables in the raw data (state table) database are converted here to unique multinomial variables.
